# Phylogenetic relationships and subgeneric classification of European *Ephedrus* species (Hymenoptera, Braconidae, Aphidiinae)

**DOI:** 10.3897/zookeys.878.38408

**Published:** 2019-10-07

**Authors:** Korana Kocić, Andjeljko Petrović, Jelisaveta Čkrkić, Milana Mitrović, Željko Tomanović

**Affiliations:** 1 University of Belgrade-Faculty of Biology, Institute of Zoology. Studentski Trg 16, 11000 Belgrade, Serbia; 2 Institute for Plant Protection and Environment, Department of Plant Pests, Banatska 33, 11000 Belgrade, Serbia

**Keywords:** molecular phylogeny, *Ephedrus* subgenera, *Ephedrus hyadaphidis* sp. nov.

## Abstract

In this study two molecular markers were used to establish taxonomic status and phylogenetic relationships of *Ephedrus* subgenera and species distributed in Europe. Fifteen of the nineteen currently known species have been analysed, representing three subgenera: *Breviephedrus* Gärdenfors, 1986, *Lysephedrus* Starý, 1958 and *Ephedrus* Haliday, 1833. The results of analysis of COI and EF1α molecular markers and morphological studies did not support this classification. Three clades separated by the highest genetic distances reported for the subfamily Aphidiinae on intrageneric level. *Ephedrus brevis* is separated from *persicae* and *plagiator* species groups with genetic distances of 19.6 % and 16.3 % respectively, while the distance between *persicae* and *plagiator* groups was 20.7 %. These results lead to the conclusion that the traditional subgeneric classification of *Ephedrus* needs revision. Species from *persicae* species group are raised to subgenus level as *Fovephedrus* Chen, 1986 and *Lysephedrus***syn. nov.** is assigned as a junior synonym of subgenus Ephedrus. Key for identification of *Ephedrus* subgenera is provided. *Ephedrus hyadaphidis* Kocić & Tomanović **sp. nov.** is described and several species are confirmed as valid species for the first time. Furthermore, two species are synonymised: *E. dysaphidis***syn. nov.** as a junior synonym of *E. cerasicola* and *E. blattnyi***syn. nov.** as a junior synonym of *E. plagiator*.

## Introduction

Members of the subfamily Aphidiinae (Hymenoptera, Braconidae) display a fascinating life cycle as obligatory koinobiont parasitoids of aphids, regulating host population size and density and therefore they are considered as a beneficial insect group. Due to their importance in biological control of aphids, this is one of the most extensively investigated groups within the family Braconidae (Quicke 2015), yet their phylogeny and taxonomy still remains unresolved.

Approximately 40 species worldwide belong to the genus *Ephedrus* (Akhtar et al. 2011); however, this number is continuously changing with the description of new species and synonyms of already described species. In Europe, this genus is represented with 19 valid species: *Ephedrus blattnyi* Starý, 1973, *E. brevis* Stelfox, 1941, *E. cerasicola* Starý, 1962, *E. chaitophori* Gärdenfors, 1986, *E. dysaphidis* Tomanović, Kavallieratos & Starý, 2005, *E. helleni* Mackauer, 1968, *E. koponeni* Halme, 1992, *E. lacertosus* Haliday, 1833, *E. laevicollis* Thomson, 1895, *E. longistigmus* Gärdenfors, 1986, *E. lonicerae* Tomanović, Kavallieratos & Starý, 2009, *E. nacheri* Quilis Perez, 1934, *E. niger* Gautier, Bonnamour & Gaumont, 1929, *E. persicae* Froggatt, 1904, *E. plagiator* Nees, 1811, *E. prociphili* Starý, 1982, *E. vaccinii* Gärdenfors, 1986, *E. validus* Haliday, 1833 and *E. tamaricis* Tomanović & Petrović, 2016. Although there are three more species which apparently inhabit Europe, *E. angustithoracicus* Kiriyak, 1977, *E. hyaloptericolus* Kiriyak, 1977 and *E. mandjuriensis* Kiriyak, 1977, these cannot be treated as valid species in absence of available type material and with species descriptions corresponding to *E. nacheri*, *E. niger* and *E. plagiator*, respectively. The distribution patterns of *Ephedrus* species are very diverse: some being distributed throughout the continent like *E. persicae* or *E. plagiator* or some being restricted to certain microhabitats, for instance, *E. lonicerae* (Tomanović et al. 2007). Furthermore, specialisation to a certain aphid host ranges from strict specialists that parasitise only one species or genus (e.g., *E. prociphili*, specialised parasitoid of the genus *Prociphilus* Koch, 1857) to broadly oligophagous (or polyphagous) species such as *E. persicae* and *E. plagiator*. Both species have been found to attack more than 100 aphid species (each) belonging to different tribes (Starý 1981, Gärdenfors 1986, Žikić et al. 2009, 2017a). Several recent studies suggest that generalist Aphidiinae species might actually not be that broadly oligophagous, but instead composed of cryptic species complexes (Mitrovski-Bogdanović et al. 2013, Derocles et al. 2016, Petrović et al. 2016).

Several species from this genus were implemented in biological control programs, and some of them are commercially produced and packed as part of the cocktails containing different parasitoid agents (Menten 2011). Two of *Ephedrus* species are commercially sold in great numbers, *E. cerasicola* (hundreds of thousands to one million of specimens per week) and somewhat less *E. plagiator* (ten thousand to hundred thousand per week) (van Lenteren et al. 2018). Moreover, there have been attempts of *Ephedrus* species introductions to different continents in order to control alien aphid species with the same geographical origin (Autrique et al. 1989, Carver 1989, Starý 1993, Elliott et al. 1995, Ripa et al. 1995, Armstrong and Peairs 1996). Unfortunately, in the majority of these introductions, *Ephedrus* populations were not established.

Systematic position of *Ephedrus* within the subfamily Aphidiinae is still uncertain. There have been numerous hypotheses about which genus or group is most related to *Ephedrus*. Based on wing venation and several other symplesiomorphic characters, the genera *Toxares* Haliday, 1833 and *Ephedrus* were classified within the tribe Ephedrini (Mackauer, 1968), yet studies on larval morphology showed that they differ significantly (Finlayson 1990). Furthermore, morphological examination of the first instar larvae indicated great morphological similarity between *Ephedrus* and *Praon* Haliday, 1833 (O’Donnell 1989). The genus *Parephedrus* Starý & Carver, 1971 from Australia is considered related to *Ephedrus* (Starý and Carver 1971; Mackauer and Finlayson 2012), sharing similar wing venation and 11 segmented antennae in both sexes. Although Schlinger (1974) positioned *Pseudephedrus* Starý, 1972 and *Parephedrus* within the subfamily Ephedrinae (now tribe Ephedrini), these two archaic genera together with *Vanhartenia* Starý and van Harten, 1974 and *Choreopraon* Mackauer, 2012 are considered to form a separate branch in the phylogeny of Aphidiinae (Starý 1976).

The second uncertainty about the systematic position of the genus *Ephedrus* is whether or not it represents the basal group within the subfamily Aphidiinae. There are some, mostly molecular, studies which consider the tribe Praini as basal within the subfamily (Smith et al. 1999), while some others listed *Aclitus* (Kambhampati et al. 2000) or even *Pseudephedrus* (Žikić et al. 2017b) as basal genera. On the other hand, numerous molecular and morphological studies suggest that *Ephedrus* is the most likely candidate for taking a basal position (Gärdenfors 1986, Belshaw and Quicke 1997, Sanchis et al. 2001, Shi and Chen 2005). Species of this genus possess a range of plesiomorphic characters, such as forewing with fully developed braconid venation, 11-segmented antennae in both sexes, existence of central areola of propodeum, shape of ovipositor sheaths, short petiole and several more (Gärdenfors 1986). Additional evidence that goes into favour of its basal position is the discovery of fossil specimens resembling species of this genus, namely *Ephedrus mirabilis* Timon-David, 1944 and *Ephedrus primordialis* Brues, 1933 (Starý 1973, Ortega-Blanco et al. 2009).

Until now, species of *Ephedrus* have only been used for molecular studies on the subfamily level (Belshaw and Quicke 1997, Smith et al. 1999, Kambhampati et al. 2000, Sanchis et al. 2001, Žikić et al. 2017b), while taxonomic status of its subgenera and relationships between species have never been tested with molecular markers. Currently, genus *Ephedrus* comprises three subgenera: *Lysephedrus* (*E. validus*), *Breviephedrus* (*E. brevis*) and *Ephedrus* (all other species), and the third is divided into three species groups (*plagiator*, *lacertosus*, *persicae*) (Gärdenfors 1986). Based on the presence of fovea on mesoscutum, Chen (1986) described the genus *Fovephedrus* Chen, which was later synonymised with *Ephedrus*, but proposed as a separate subgenus (van Achterberg 1997). However, position of *Fovephedrus* within genus *Ephedrus* was completely unclear.

We decided to investigate the taxonomic status and phylogenetic relationships of *Ephedrus* subgenera and species with European origin, with the combination of nuclear and mitochondrial markers and morphology. Here we propose a new subgeneric classification. Several species in this study are confirmed by molecular approach for the first time and phylogenetic relationships among European species of genus *Ephedrus* are presented. We describe *Ephedrus hyadaphidis* sp. nov., an additional member of the *plagiator* group, parasitoid of *Hyadaphis foeniculi* Passerini, 1860 on several plant species. Furthermore, we synonymise *E. dysaphidis* as a junior synonym of *E. cerasicola* and *E. blattnyi* as a junior synonym of *E. plagiator*.

## Materials and methods

### Sample collection and morphological analysis

Parasitoid specimens were sampled throughout Europe during the past two decades. Sampling was conducted in two ways, by net sweeping and by rearing of the parasitoids. The second method was preferred as rearing provides important data on their tri-trophic interactions. Parts of the plants infested with aphid colonies were stored in plastic containers with the lid openings covered with mesh. The samples were transported to laboratory and kept under controlled conditions until the emergence of the parasitoids. Aphids stored in 70% ethanol and plant samples were also collected and identified to species or genus level. Unfortunately, all specimens of *E. lonicerae* were slide mounted and thus couldn’t be used for molecular analyses (Žikić et al. 2009) and after a thorough search, we were not able to locate and acquire specimens of *E. vaccinii* or *E. longistigmus*.

Each specimen was examined under the ZEISS Discovery V8 stereomicroscope (Carl Zeiss MicroImaging GmbH, Gottingen, Germany). After DNA extractions, samples were dissected and slide-mounted in Berlese medium. When available, specimens were studied using the Jeol JSM–6460LV scanning electron microscope (Jeol USA, Inc., Peabody, MA, USA). Measurements for new species description were obtained using ImageJ software (Schneider et al. 2012) based on photographs taken with Leica DM LS phase contrast microscope (Leica Microsystems GmbH, Wetzlar, Germany). Morphological terminology follows Sharkey and Wharton (1997).

The examined material is deposited in the Insitute of Zoology, Faculty of Biology, University of Belgrade (Serbia), except for specimens of *E. validus* and *E. koponeni* that were loaned from the Zoological Museum, University of Helsinki (Finland) and 5♀9♂ of *E. hyadaphidis* paratypes that are deposited in the Croatian Natural History Museum, Zagreb, Croatia. *Ephedrus* specimens analysed in this study are presented in Suppl. material 1.

### Molecular analysis

Total genomic DNA was extracted from single individuals using the Qiagen Dneasy Blood & Tissue Kit (Qiagen, Valencia, CA, USA), following the manufacturers’ protocol. The extraction method was non destructive in order to preserve whole specimens that could be used in further studies of external morphology. Two molecular markers were used; mitochondrial cytochrome c oxidase subunit I (COI) and nuclear elongation factor 1 alpha (EF1α). The primers used for amplification of COI and EF1α fragments were LCO1490 and HCO2198 (Folmer et al. 1994) and EFf and EFr (Belshaw and Quicke 1997), respectively. The amplification was carried out in a total volume of 40 µl which contained 2 µl of extracted DNA, 0.4 µl of Taq polymerase, 2 µl of each primer (10 mM), 2.4 µl of dNTPs (0.6 mM), 3.6 µl of MgCl_2_, 4 µl of buffer and 23.6 µl of nuclease free water. PCR profile for COI was: 5 minutes of initial denaturation followed by 35 cycles of 60 second denaturation (94 °C), 60 second annealing (54 °C) and 90 second extension (72 °C) and 7 minutes of final extension (72 °C). PCR conditions for amplification of EF1α were same as in Belshaw and Quicke (1997). Obtained PCR products were purified with QIAquick PCR Purification Kit (Qiagen, Valencia, CA, USA) and sent to Macrogen Inc. (Seoul, Korea) for sequencing. DNA of dry museum specimens, due to fragmentation of DNA, was amplified by using primers for amplification of short barcoding fragments, following the protocol by Mitrović and Tomanović (2018). For certain specimens the quantity of extracted DNA was too low, so only the COI fragment was obtained. That was the case with *E. brevis* individuals. For others, like *E. chaitophori* and *E. tamaricis*, the amplification of EF1 alpha fragment yielded PCR products of high quality, but the sequencing was unsuccessful. The reason for this might be the interruption of sequencing due to the existence of DNA introns or a deletion along the fragment.

### Phylogenetic analysis

The sequences acquired after amplification were checked for pseudogenes, visualised in FinchTV Geospiza Inc. (Seattle, USA) and manually edited and aligned in BioEdit program (Hall 1999). Sequences were trimmed to the same length of 557 and 440 base pair positions for COI and EF1α, respectively. MEGA 6 (Tamura et al. 2013) software was used to estimate the evolutionary divergence between sequences. The analysis for both gene fragments was conducted independently in BEAST 2.5 (Drummond et al. 2012) software platform for Bayesian evolutionary analysis. The initial data file was designed in BEAUti with the Tamura-Nei (Tamura and Nei 1993) model with gamma distributed rates among sites (TN93+G), which MEGA 6 proposed as the most appropriate model. The initial data file conducted the analysis with strict clock type and Yule Process speciation. The species *Venturia canescens* Gravenhorst, 1829, belonging to the sister family Ichneumonidae was used as an outgroup species in all analyses. Obtained phylogenetic trees which contained all sequences visualised by FigTree 1.4.3. software (Rambaut 2009) were large and hard to follow, thus a single sequence of each haplotype was used to present the results.

## Results

**Figure 1.**
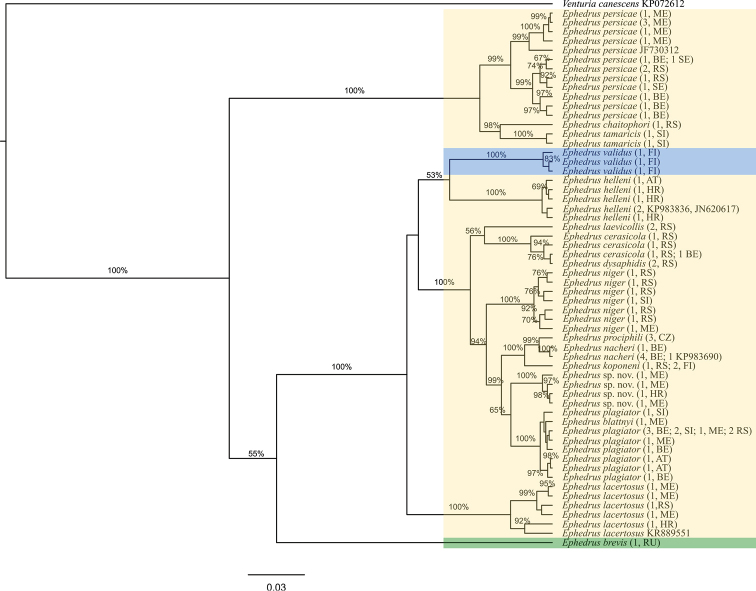
Bayesian inference phylogram for cytochrome oxidase *c* subunit I mitochondrial sequences. Bayesian posterior probabilities above 50 % are shown. The traditional subgenera are marked in different colours: *Lysephedrus* (blue), *Breviephedrus* (green), and *Ephedrus* (yellow). The number of sequences with the same haplotype and countries of origin are presented in brackets. Country abbreviations: AT – Austria, BE – Belgium, CZ – Czech Republic, FI – Finland, HR – Croatia, ME – Montenegro, RS – Republic of Serbia, RU – Russia, SI – Slovenia.

**Figure 2.**
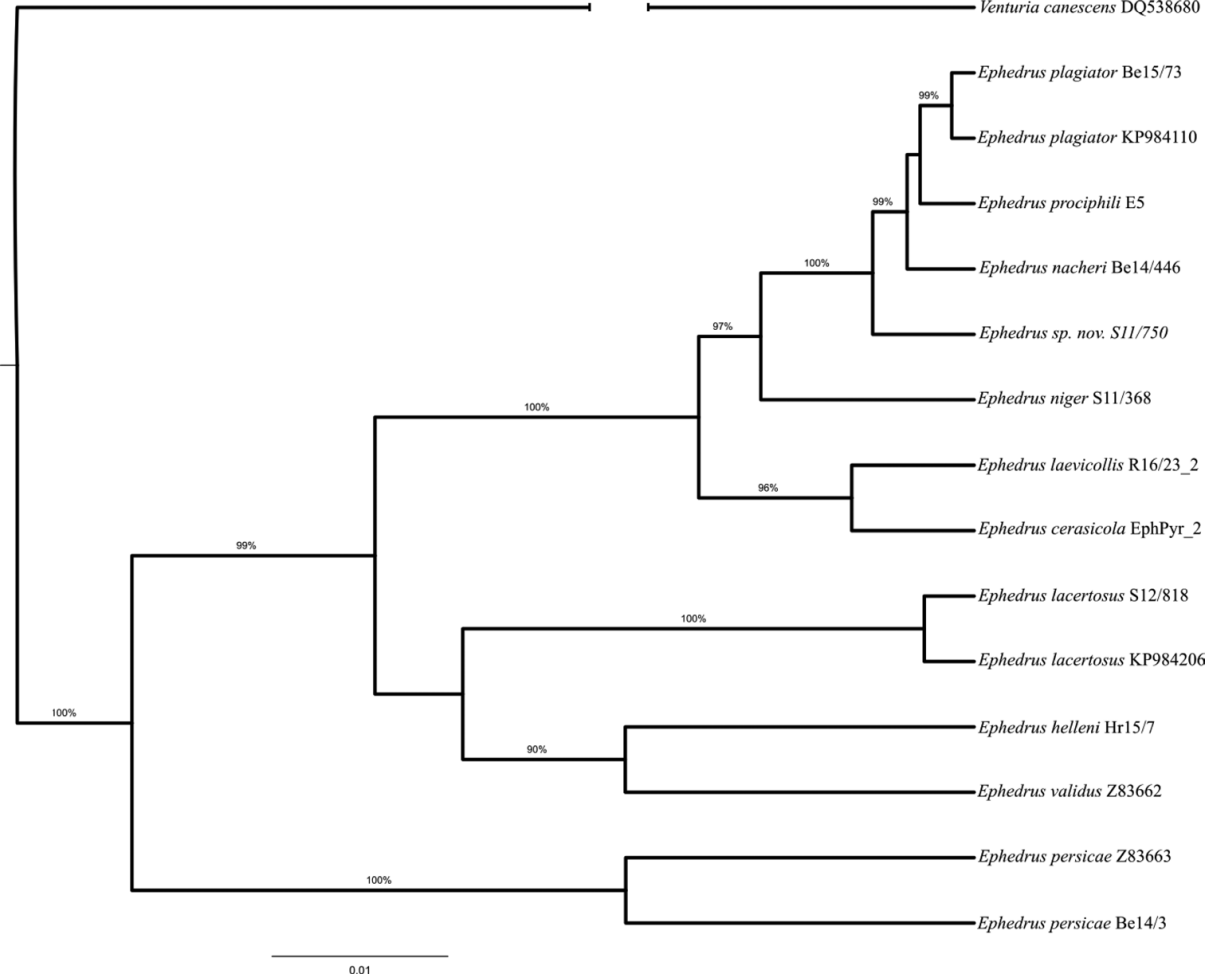
Bayesian inference phylogram for elongation factor 1α nuclear sequences. Bayesian posterior probabilities above 50 % are shown.

Phylogenetic analyses of the two molecular markers yielded trees with similar branch topographies (Figs 1, 2). A total of 86 COI sequences was analysed (seven, including outgroup, were mined from GenBank and 18 were used from authors’ previous publications), and 58 different haplotypes were detected (Fig. 1). Species *E. brevis*, *E. cerasicola*, *E. laevicollis*, *E. validus*, and *E. prociphili* are confirmed as distinct species for the first time with the barcoding marker. Phylogenetic tree based on COI sequences showed species separation into three main phylogenetic clades, which does not correspond to traditional subgenera delineation (Fig. 1). First clade was formed by species belonging to *persicae* species group (*E. persicae*, *E. chaitophori*, and *E. tamaricis*). Second clade was represented with *E. brevis* and third with all other species (*plagiator* clade). *Ephedrus brevis*, the only representative of Breviephedrus subgenus was separated from both persicae and *plagiator* clades on the phylogenetic tree with the average genetic distances of 19.6 % and 16.3 %, respectively, while genetic distance between *persicae* clade and *plagiator* clade was 20.7 % (Suppl. material 2).

According to the obtained results, the nominative subgenus Ephedrus is paraphyletic, consisting of two independent lineages, *plagiator* and *lacertosus* species groups. In addition to these groups *E. validus*, the only member of the *Lysephedrus* subgenus, also grouped within this clade. Moreover, *E. validus* is nested within the *plagiator* species group, forming a separate clade together with *E. helleni*. The second clade within the *plagiator* species group consists of specimens belonging to ten different species (*Ephedrus blattnyi*, *E. cerasicola*, *E. dysaphidis*, *E. hyadaphidis* sp. nov., *E. koponeni*, *E. laevicollis*, *E. nacheri*, *E. niger*, *E. plagiator*, and *E. prociphili*). One individual determined as *E. blattnyi* grouped with *E. plagiator* specimens, with the genetic distance ranging from 0.2 % to 0.7 %. The same was the case for one *E. dysaphidis* specimen which clustered within the *E. cerasicola* clade (genetic distance 0.0 %–1.6 %). Genetic distances between other species within this group vary greatly ranging from 1.1 % between *E. nacheri* and *E. prociphili* up to 7.6 % between the previous two and *E. laevicollis*. *Ephedrus lacertosus* is clearly separated as a distinct group with genetic distances from all other *Ephedrus* species above 8.9 %. The clade consisting of *Ephedrus persicae* species is the oldest within the genus and represents a separate subgenus. Within this clade all three analysed species were confirmed as valid. Additionally, within *E. persicae* two separate phylogenetic lines were determined with average genetic distance of 2.5 % (Fig. 1).

With the second molecular marker EF1α, 14 sequences (four mined from GenBank), belonging to eleven species (*Ephedrus cerasicola*, *E. helleni*, *E. hyadaphidis* sp. nov., *E. lacertosus*, *E. laevicollis*, *E. nacheri*, *E. niger*, *E. persicae*, *E. plagiator*, *E. prociphili*, *E. validus*) were analysed. While the genetic distances between species groups and species were significantly lower than those based on COI sequences, the topography of the phylogenetic three remained similar (Fig. 2). As previously, sequences separated into two groups, the first composed solely out of the representative of *persicae* species group, *E. persicae*, and the second containing species from *lacertosus* and *plagiator* groups. A significant change in relation to the COI phylogenetic tree is clustering of *E. validus* and *E. helleni* together with *E. lacertosus*, species from *lacertosus* group. This topography could be explained by genetic distances: *E. helleni* and *E. validus* show genetic distances of 3.8–7.2 % and 5.7–9.0 %, respectively, when compared to other *plagiator* species, whereas compared to *lacertosus*, these distances are 4.2 % and 5.4 %, respectively. All genetic distances were considerably lower compared to those based on COI, since EF1α is more conservative nuclear molecular marker (see Suppl. material 2). The average genetic distances between *E. persicae* and *lacertosus* and *plagiator* species groups were 7.6 % and 9.0 %, respectively.

The analysis of the COI molecular marker grouped sequences designated as *Ephedrus* sp. nov. into a distinct clade. Genetic distance parameters position sequences of this group as most closely related to *E. plagiator* (2.7 %–3.0 %) and other members of *plagiator* species group (4.3 %–8.0 %). Results of the EF1α phylogenetic analysis confirmed the position of these sequences in relation to other species.

Substantial morphological examination of all available specimens of genus *Ephedrus* resulted in discovery of one species new to science, designated as *Ephedrus* sp. nov. in the phylogenetic analysis.

### Description of the new species

#### Ephedrus hyadaphidis

Taxon classificationAnimaliaHymenopteraBraconidae

Kocić & Tomanović sp. nov.

E17E6221-C77B-5037-BEB2-0B5616B55524

http://zoobank.org/92D3A33E-42A9-4177-98DC-40B13653F28D

Fig. 3

##### Material.

Holotype ♀ from Montenegro: Durmitor-Sušica, 27.07.2012, reared from *Hyadaphis foeniculi* on *Sanicula europaea*. Paratypes: 2♂ (slide mounted) from Montenegro: Durmitor-Sušica, 27.07.2012, reared from *Hyadaphis foeniculi* on *Sanicula europaea*. 3♀6♂ from Montenegro: Crno jezero, 20.06.2004, from *Hyadaphis foeniculi* on *Lonicera xylosteum*. 5♂ from Montenegro: Durmitor-Sušica, 22.07.2004, reared from *Hyadaphis foeniculi* on *Sanicula europaea*. 5♀9♂ (2♀ mounted) from Croatia: Plitvička jezera-Milanovac, 20.06.2015, from *Hyadaphis foeniculi* on *Anthriscus sylvestris*.

##### Diagnosis.

The new species belongs to *E. plagiator* species group, due to fore wing venation. It is differentiated from other *Ephedrus* species by possessing considerably short first flagellar segment; F1 is 2.40–2.65 as long as wide (the closest ratio is in *E. nacheri*, 3.05–3.7). The new species is most closely related to *E. plagiator.* Beside the shorter F1, it can be distinguished from *E. plagiator* by a smaller number of longitudinal placodes on F2 (2–3 compared to 4–6) and wider pterostigma (4.15–4.35 compared to 4.4–4.75 in *E. plagiator*). The new species is a specialised parasitoid of *Hyadaphis foeniculi*, occurring in the Balkan Peninsula.

##### Description.

**Figure 3.**
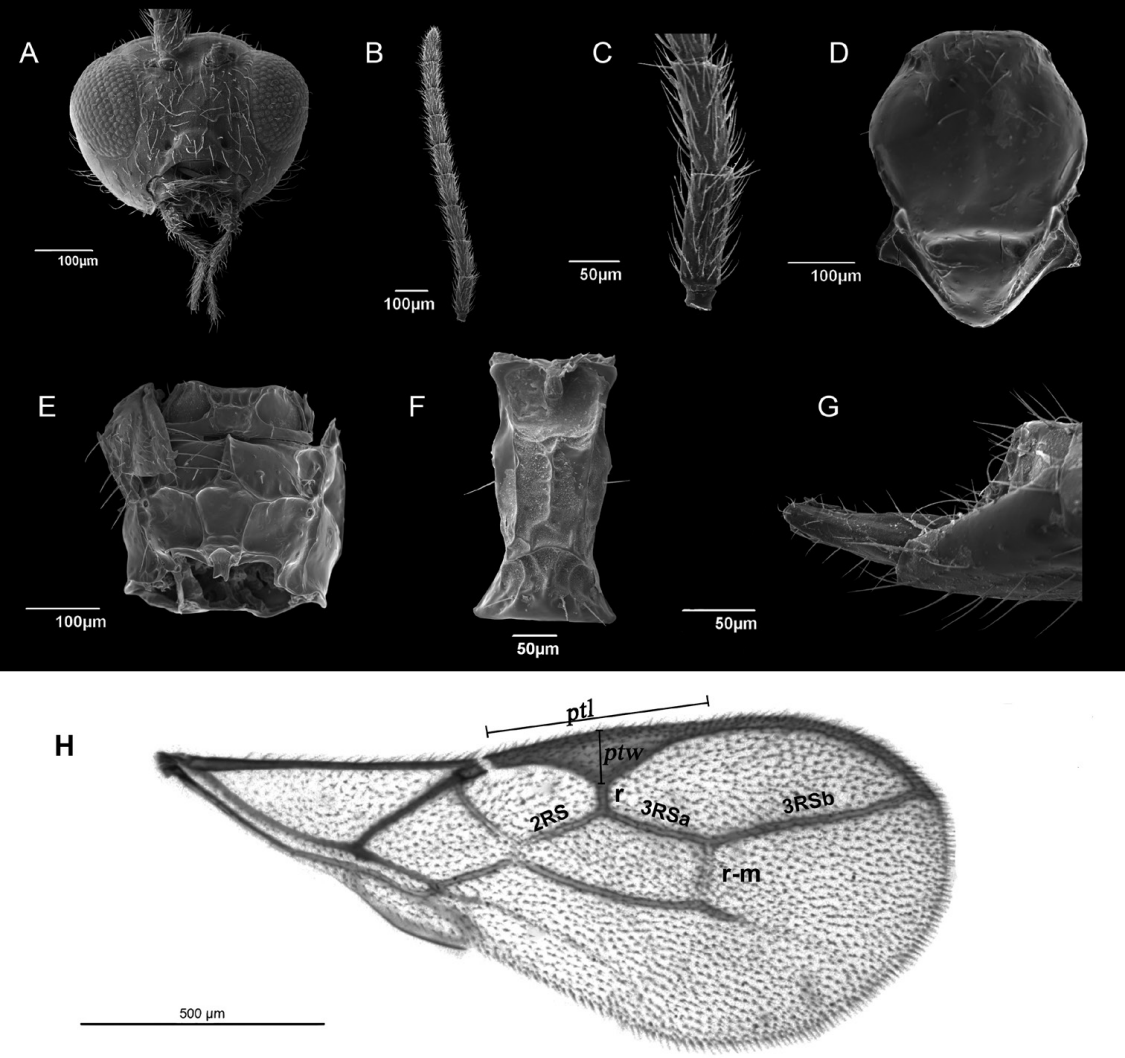
*Ephedrus hyadaphidis* Kocić & Tomanović, sp. nov., female, scanning electron microscopy **A** Head, anterior view **B** Antennae, lateral view **C** First and second antennal segments, lateral view **D** Mesoscutum, dorsal view **E** Propodeum, dorsal view **F** Petiole, dorsal view **G** Ovipositor sheaths, lateral view **H** Forewing, with designated vein terminology. Abbreviations: ptl – pterostigma length, ptw – pterostigma width.

*Female.***Head** (Fig. 3A). Eyes medium sized, oval, prominent and sparsely haired. Clypeus somewhat convex, bearing eight long setae. Frons with medium number of setae. Tentorial index 0.6–0.7. Tentorial pits deep. Malar space to longitudinal eye diameter ratio 0.4. Maxillary palps with four palpomeres, labial with two, all of them densely setose. Antennae 11–segmented, filiform, with semi-erect setae that are subequal to 2/3 of the flagellar segment diameter (Fig. 3B). First flagellar segment (F1) 2.41–2.67 times as long as wide, bearing 2–3 longitudinal placodes (Fig. 3C). F2 2.31–2.65 times as long as wide, with 2–3 longitudinal placodes. Number of longitudinal placodes on the remaining seven flagellar segments remains low (F3 2–4, F4 2–4, F5 3–5, F6 3–6, F7 4–6, F8 4–6, F9 4–6). F8 and F9 separated, but due to dense hairs may not seem that visible. **Mesosoma**. Mesoscutum with slightly crenulated notaulices distinct only in anterior part (Fig. 3D). Along mesoscutum two rows of sparse setae present. Propodeum areolated with regular carinae and pentagonal central areola (Fig. 3E). Upper and lower areolae with 2–4 setae. **Forewing** (Fig. 3H). Forewing length 1.6 mm, width 0.6 mm. Pterostigma 4.15–4.35 as long as wide. Pterostigma width to r vein ratio (ptw/r) 1.62–1.87. 3Rsa/r-m and 3Rsb/3Rsa vein ratios 1.62–1.70 and 1.97–2.14, respectively. Vein 2Rsa not visible in first third, therefore may appear shorter than it is. 3Rsa and 2Rsa vein ratio 1.1–1.2. **Metasoma.** Petiole slender, 2.05–2.15 as long as wide at spiracle level (Fig. 3F). Central carina is prominent, while dorso-lateral carinae are slightly distinct. Posterior lateral excavations visible. Ovipositor sheaths elongated, 3.3 times as long as wide, bearing sparse setae along the surface (Fig. 3G). **Colour**. Head and mesoscutum brown, the rest of the mesosoma and petiole yellowish brown. Mouthparts brown. Scape and pedicel brown, F1 with yellow ring at the base, remaining flagellar segments brown. Metasoma and ovipositor sheets brown. Legs yellowish. Body length 1.8 mm. *Male.* Eyes slightly more convex than in female. Head with sparse hairs. Tentorial index 0.45. Malar space to longitudinal eye diameter ratio 0.3. Antennae 11–segmented, with long setae along the surface, almost equal to flagellum diameter. F1 and F2 2.45 times as long as wide, both with 2–3 longitudinal placodes. F2 subequal to F1. Maxillary palps with four palpomeres, labial with two. Mesoscutum like in female. Propodeum with pentagonal central areola and regular carinae. Pterostigma slightly wider than in female, with the length to width ratio 3.8–3.9. Vein ratios Ptw/r, 3Rsa/r-m and 3Rsb/3Rsa are 1.8–1.9, 1.85–1.90 and 1.8–1.95, respectively. 3Rsa to 2Rsa vein ratio is 1.3–1.4. Petiole 2 times as long as wide at spiracle level. Petiole with visible central and lateral carinae. **Colour.** Same as in female.

##### Etymology.

Name of the species is derived from its aphid host, *Hyadaphis foeniculi*.

##### Distribution.

The current species distribution is Balkan Peninsula.

##### Depositories.

Holotype is slide mounted and deposited in the collection of the Institute of Zoology, Faculty of Biology, University of Belgrade. Paratypes collected in National Park Plitvice, Croatia are deposited in the Croatian Natural History Museum, Zagreb, Croatia. The remaining paratypes are deposited at the Institute of Zoology, Faculty of Biology, University of Belgrade.

##### Synonymies.

The molecular phylogenetic analysis clustered *E. dysaphidis* together with *E. cerasicola*, with the genetic distance ranging from 0.0% to 1.6%. We performed a detailed morphological examination of all available material of both species in order to test the obtained molecular results. *Ephedrus dysaphidis* is a species from *plagiator* species group, described in the study by Tomić et al. (2005). Authors differentiated it from other species in this group by number of longitudinal placodes on F1 (1–2, rarely 3) and F2 (2–3, rarely 4) and shorter petiole (1.94–2.20 times as long as wide). The colour of scapus, pedicel and the ring at the base of F1 is stated as brownish to yellow. It is a parasitoid of aphids from genus *Dysaphis* Börneron on *Malus domestica* Borkh. While studying morphology of many additional populations of *E. dysaphidis* and *E. cerasicola*, we found numerous overlapping characters. In the description of *E. cerasicola*, Starý (1962) states that F1 is more than three times as long as wide, while in the paper of Tomić et al. (2005) the length/width ratio of *E. dysaphidis* is 3.8–4.6. Furthermore, F1/F2 length ratio is also overlapping in both species (in *E. cerasicola* F1 is longer by 1/3 than F2 and in *E. dysaphidis* that ratio is 1.28). Propodeum in both species is with a pentagonal areola, having the same number of setae on upper and lower areolae. Additionally, pterostigma in *E. cerasicola* is more than four times as long as wide; in *E. dysaphidis* this ratio varies between 4.1–4.7. Finally, petiole in *E. cerasicola* is less than twice as long as wide, while in *E. dysaphidis*, according to the authors, this ratio is 1.94–2.20. However, our studied material of both species showed that the length to width ratio at the spiracle level of *E. cerasicola* and *E. dysaphidis* is 1.97–2.1 and 1.90–2.18, respectively, thus also overlapping. The only character that differs in two descriptions of species is the number of longitudinal placodes on F1 and F2 flagellar segments, which is 0 and 2 in *E. cerasicola* and, as mentioned above, 1–2(3) and 2–3(4) in *E. dysaphidis*, respectively. However, we found specimens from the same sample (that were first identified as *E. dysaphidis* based on the aphid host *Dysaphis plantaginea* Passerini, 1860 on *Malus domestica*) having a different number of longitudinal placodes that varied from F1:0, F2:1 to F1:1, F2:3. Moreover, in the revision of the genus *Ephedrus*Gärdenfors (1986) states that the number of longitudinal placodes in F1 and F2 for *E. cerasicola* is 1 and 2–3, respectively. One more distinction between the species was the colour of scape, pedicel and F1 and F2. In *E. cerasicola* scape, pedicel, F1 and part of F2 are yellow brownish (Starý 1962) or can be with scape yellowish to brown, pedicel and at least base or the entire F1 yellow, while F2 is yellowish at base (Gärdenfors 1986). While examining the material of both species we found various gradations of scape, pedicel, F1 and F2 colour, mainly following the description of Starý (1962) and Gärdenfors (1986). Although important morphological variability exists within *E. cerasicola* host associated lineages, after examining all the available data we here synonymise *E. dysaphidis* as a junior synonym of *E. cerasicola*.

Material examined. ***E. dysaphidis***: Holotype ♀ from Serbia: Belgrade, 08.05.1995, reared from *Dysaphis* sp. on *Malus domestica*. Paratypes 3♀ from Serbia: Belgrade, 08.05.1995, reared from *Dysaphis* sp. on *Malus domestica*. 1♀ from Serbia: Belgrade-Radmilovac, 22.04.1992, reared from *Dysaphis* sp. on *Malus domestica*. 3 ♀ Serbia: Belgrade-Zemun, 22.04.2014, from *Dysaphis devecta* Walker on *Malus* sp. 2 ♀ Serbia: Belgrade, 24.04.2014, from *Dysaphis devecta* on *Malus* sp. ***E. cerasicola***: 1♀ from Montenegro: Zminje jezero, 04.08.1982. 1♀ Serbia: Belgrade-Crveni krst, 14.06.1997, from *Myzus cerasi* Fabricius 1775 on *Prunus cerasus* L. 1♀ from Serbia: Mladenovac, 11.06.1990. 1♀ from Serbia: Belgrade-New Belgrade, 17.06.1993, from *Phorodon humuli* Schrank, 1801 on *Prunus cerasifera* 1♀ from Serbia: Kopaonik, 05.07.1997, from *Brachycaudus helichrysi* Kaltenbach, 1843 on *Myosotis* sp. 2♀1♂ from Serbia: Subjel, 01.05.2017., from *Dysaphis pyri* Boyer de Feonscolombe, 1841 on *Pyrus communis*. 4♀ from Belgium: from *Dysaphis plantaginea* on *Malus* sp.

*Ephedrus blattnyi* is a specialised parasitoid described from one finding in the Czech Republic, reared from *Pterocomma ringadhli* (junior synonym of *Pterocomma rufipes* Hartig, 1841) on *Salix caprea*. Several authors during previous years questioned the validity of *E. blattnyi* species status. After the examination of type specimens Gärdenfors (1986) stated that they are extraordinarily similar to *E. plagiator* and that it is possible that they represent aberrant specimens of *E. plagiator* due to an unusual aphid host. Furthermore, Koponen and Halme (1993) reported that they collected specimens fully corresponding to the *E. blattnyi* redescription (Gärdenfors, 1986). However, the authors didn`t include them in the paper, since the distinguishing characters were not reliable. Finally, Tomanović (2000) states, while reporting the finding of specimens corresponding to *E. blattnyi*, that it is very similar to *E. plagiator* and *E. prociphili*. We analysed specimens from *P. rufipes* which morphologically corresponded to the description of *E. blattnyi* reared from *P. rufipes* aphid host on *Salix retusa*. Molecular analysis of COI clustered *E. blattnyi* within the *E. plagiator*, with the molecular distance ranging from 0.2% to 0.7%. Therefore, we conclude that *E. blattnyi* represents a morphotype of *E. plagiator* and assign it a status of junior synonym.

### Subgenera and species groups of the genus *Ephedrus* in Europe

Analysing all obtained results (both molecular and morphological), we concluded that the current subgeneric classification of *Ephedrus* needs revision and here we propose a new one. Subgenus LysephedrusStarý 1958 is synonymised as a junior synonym of the subgenus Ephedrus Haliday, 1833 from which *persicae* species group is raised to the level of the subgenus Fovephedrus Chen, 1986. The subgenera *Fovephedrus* and *Ephedrus* are redescribed.

#### Subgenus Fovephedrus

Taxon classificationAnimaliaHymenopteraBraconidae

Chen, 1986

0C04A02A-6E11-56A4-BCE7-B535FC511E64

##### Type species.

*Fovephedrus radiatus* Chen, 1986.

##### Etymology.

Name derived from presence of fovea on mesoscutum of type species

##### Diagnosis.

3Rsa vein shorter than 2Rsa (3Rsa / 2Rsa less than 1), petiole subquadrate.

##### Description.

F1 with smaller number of longitudinal placodes (0–3, rarely 4). Mesoscutum usually with one or two mesoscuteal foveae, sometimes lacking both. Scutellar sulcus always undivided. 3RSa shorter than 2Rsa (0.55–0.95). 3Rsb/3Rsa 2.65–3.4. Petiole short and broad, 1.3–1.5 times as long as wide, lacking post lateral excavations. Ovipositor sheaths varying from stout to slender.

Species in Europe: *Ephedrus persicae* Froggatt, 1904, *Ephedrus chaitophori* Gärdenfors, 1986, *Ephedrus lonicerae* Tomanović, Kavallieratos & Starý, 2009, *Ephedrus tamaricis* Tomanović & Petrović, 2016

#### Subgenus Breviephedrus

Taxon classificationAnimaliaHymenopteraBraconidae

Gärdenfors, 1986

48634B83-1E8C-5299-BD9E-5125684D4955

##### Notes.

For diagnosis and description see Gärdenfors (1986).

**Species.***Ephedrus brevis* Stelfox, 1941.

#### Subgenus Ephedrus

Taxon classificationAnimaliaHymenopteraBraconidae

Haliday, 1833

A9FF1595-5C87-561A-B7E5-4CE9628A9ADA

Lysephedrus Starý, 1958 syn. nov.

##### Diagnosis.

3Rsa longer or subequal to 2Rsa, petiole more or less elongated.

##### Description.

F1 with variable number of longitudinal placodes (0–5). Mesoscutum without mesoscuteal fovea, except in *E. longistigmus*, and rarely *E. lacertosus*. Scutellar sulcus undivided. 3Rsa varying from almost subequal to considerably longer compared to 2Rsa (1.1–1.5 times). 3Rsb/3Rsa 1.7–2.6. Propodeum and petiole in most species without rugosity, in *E. validus* very rugose. Petiole more or less elongated, with or without post lateral excavations. Ovipositor sheaths commonly slender and elongated, in some species stout and short.

##### The *plagiator* species group.

*Ephedrus cerasicola* Starý, 1962; *Ephedrus helleni* Mackauer, 1968; *Ephedrus koponeni* Halme, 1992; *Ephedrus laevicollis* (Thomson, 1895); *Ephedrus nacheri* Quilis Perez, 1934; *Ephedrus niger* Gautier, Bonnamour & Gaumont, 1929; *Ephedrus plagiator* (Nees, 1811); *Ephedrus prociphili* Starý, 1982; *Ephedrus vaccinii* Gärdenfors, 1986; *Ephedrus validus* Haliday, 1833, *E. hyadaphidis* sp. nov.

##### The *lacertosus* species group in Europe.

*Ephedrus lacertosus* (Haliday, 1833) and *E. longistigmus* Gärdenfors, 1986.

### Key to the subgenera of European *Ephedrus*

**Table:** 

1	Scutellar sulcus divided into two (Fig. 4A), 3Rsa very long (Fig. 4C)	*** Breviephedrus ***
–	Scutellar sulcus undivided (Fig. 4D, G), 3Rsa longer or shorter than 2RS (Fig. 4F, I)	**2**
2	3Rsa visibly or slightly shorter than 2RS (Fig. 4F), petiole subquadrate (Fig. 4E)	*** Fovephedrus ***
–	3Rsa longer or subequal to 2Rs (Fig. 4I), petiole elongated (Fig. 4H)	*** Ephedrus ***

**Figure 4.**
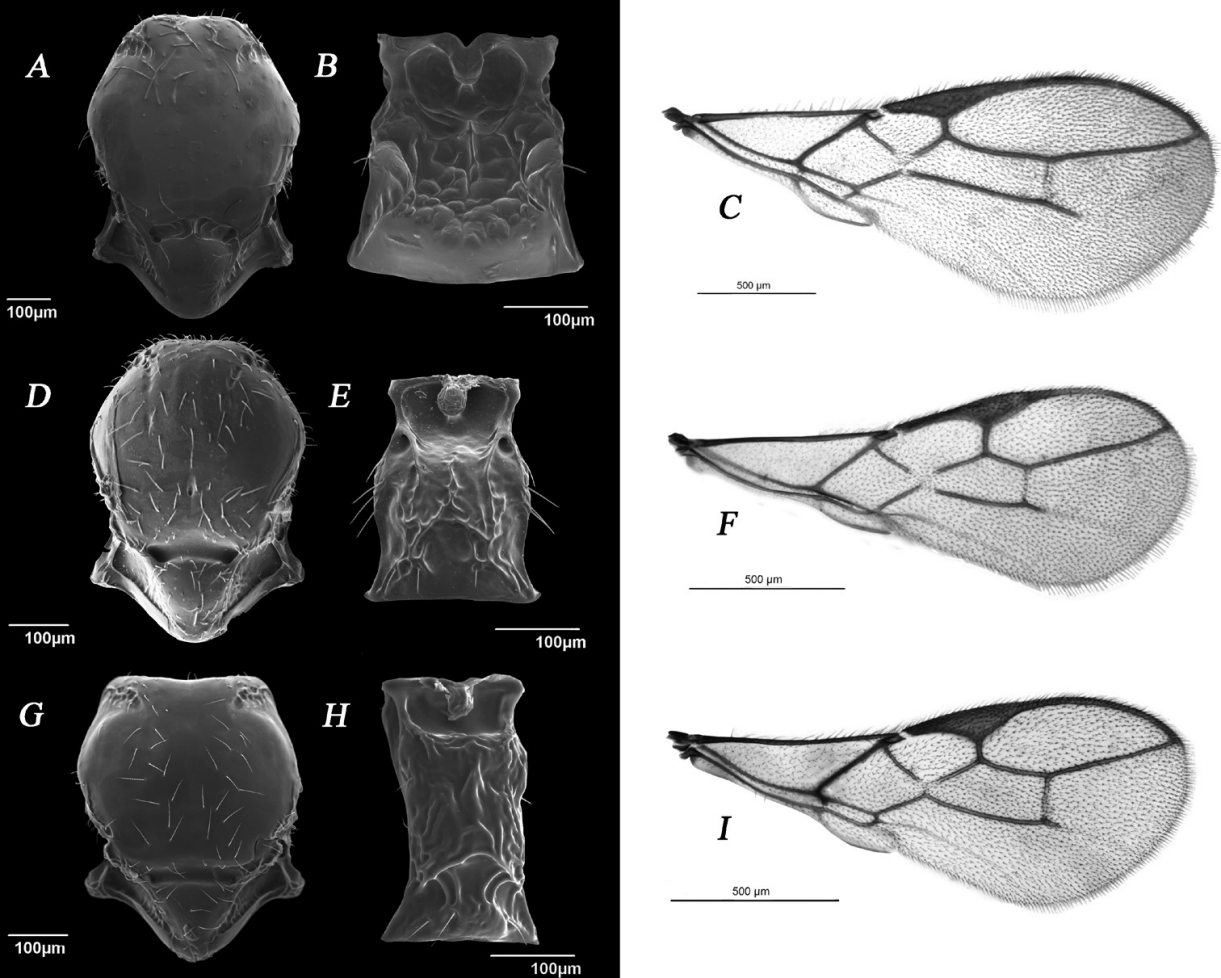
Representative species of the subgenera *Breviephedrus* (*E. brevis*), *Fovephedrus* (*E. persicae*), and *Ephedrus* (*E. plagiator*). **A–C***Breviephedrus***A** mesoscutum dorsal view **B** petiole, dorsal view **C** forewing **D–F***Fovephedrus***D** mesoscutum dorsal view **E** petiole, dorsal view **F** forewing **G–I***Ephedrus***G** mesoscutum, dorsal view **H** petiole, dorsal view **I** forewing.

## Discussion

Continuously advancing molecular methods allow us to easily sequence gene fragments of interest and compare them with results of morphological and ecological analyses in order to obtain a clearer picture of phylogenetic relationships and evolution of the group of interest. In this study we used two molecular markers to reveal the taxonomic positions and phylogeny of species within the genus *Ephedrus* in Europe. As proposed by Derocles et al. (2012), we used a combination of both mitochondrial and nuclear markers. The barcoding region of mitochondrial cytochrome c oxidase subunit I proved to be a reliable marker in resolving taxonomic relationships in previous studies of the subfamily Aphidiinae (Folmer et al 1994, Petrović et al. 2016, Tomanović et al. 2018, Mitrović et al. 2019). Furthermore, we conducted an analysis of nuclear EF1α that mostly supported the inter-specific relationships of our taxa previously provided by COI.

Previous studies classified species from genus *Ephedrus* into three subgenera (*Ephedrus*, *Breviephedrus* (*E. brevis*) and *Lysephedrus* (*E. validus*)) based on morphology (Gärdenfors 1986). However, with the combination of molecular and morphological approach we here revise the subgeneric classification. All three revised subgenera separated with the highest genetic distances known in Aphidiinae when analysing subgenera, ranging from 16.3 % to 20.7 %. Currently, the only member of the subgenus Breviephedrus, *E. brevis*, is at first sight easily distinguished from any other *Ephedrus* species with a very thickset, stocky and black polished body (Stelfox 1941). It is interesting to note that the parasitoid has never been reared from an aphid host (which is still unknown), but is suspected to be from the aphid species associated with *Betula*, since the specimens are found by sweeping or by traps in the vicinity of birch trees. Gärdenfors (1986) states this species as the most primitive within the genus, morphologically similar to *Parephedrus*. Indeed, *E. brevis* possesses a wide range of morphological characters that are also found in *Parephedrus*, thus indicating that these two could be a “connection bridge” in the evolution of ancient genera *Parephedrus*, *Vanhartenia*, *Pseudephedrus*, and *Choreopraon* on one side and the rest of Aphidiinae on the other. In order to uncover phylogenetic relationships between these groups, future studies are needed, especially in the light of our molecular results which do not support previous statements that it is the most primitive species within the genus.

Based on the presence of the fovea on mesoscutum, Chen (1986) described the genus *Fovephedrus*, with the type species *Fovephedrus radiatus* Chen, 1986. Several *E. persicae* morphotypes (*E. persicae*, *E. palaestinensis* (= *E. persicae*, see Gärdenfors (1986)), *E. rugosus*, *E. radiatus*, *E. transversus*) and *E. longistigmus* (member of *lacertosus* species group) were later reclassified to this genus, based on the same morphological character (Chen and Shi 2001). Van Achterberg (1997), synonymised *Fovephedrus* as a synonym of *Ephedrus*, but stated that it could be considered as a valid subgenus. The presence of mesoscutellar fovea is characteristic of several *Ephedrus* species. Even within *E. persicae* its presence is very variable: fovea can be absent, or the specimens can possess one (the most common state) or two foveal pits. Furthermore, this morphological character is shared with the *lacertosus* species group, where it can sometimes be found in *E. lacertosus* specimens, and is always present in *E. longistigmus*. In the end, the existence of the mesoscuteal fovea is not a character specific for species of genus *Ephedrus*, but is also found among *Toxares* (Tomanović et al. 2008). We consider this morphological character to be diagnostically unreliable, especially at the genus level.

The redescribed subgenus Fovephedrus is raised from *persicae* species group and its inter-specific relationships are thoroughly discussed in authors’ recent study (Petrović et al. 2016). We did not examine type species *E. radiatus* but judging by the description and drawings (Shi and Chen 2001) it obviously belongs to *Ephedrus persicae* species group. The same is the case with Asiatic species *E. rugosus* and *E. transversus* which on the basis of descriptions both belong to *persicae* group. As mentioned earlier, *E. palaestinensis* is synonymised with *E. persicae* (Gärdenfors 1986) and *E. longistigmus* apparently does not belong to subgenus Fovephedrus, but *lacertosus* group within the traditional subgenus Ephedrus. The subgenus Ephedrus holds the remainder of the species and is divided into two species groups, *plagiator* and *lacertosus*. While *E. longistigmus* is reported only two times (see Koponen and Halme 1993, Davidian 2018), *E. lacertosus* is common representative of the lacertosus group in Europe. This species group is considered to be the most phylogenetically advanced within the genus *Ephedrus* (Gärdenfors 1986), due to several apomorphic morphological traits, like an elongated petiole, flagellomere 1 and pterostigma. Our results confirm the separation of *E. lacertosus* into a distinct clade. Within the *plagiator* group, *E. helleni* and *E. validus* position as most distant phylogenetically from all other *plagiator* species. Both parasitoids are specialists; first one attacking *Cavariella* aphids across its distribution (Koponen and Halme 1993, Kavallieratos et al. 2004, Rakhshani et al. 2007, Starý and Havelka 2008, Tomanović et al. 2009) and additionally *Eumyzus* Shinji, 1929 in Asia (Davidian 2007, Davidian and Gavrilyuk 2014) and the second one parasitising root aphids from the subfamily Eriosomatinae (Gärdenfors 1986). Starý (1958) raised *E. validus* to the subgenus Lysephedrus, based on morphological characters, such as very rugose propodeum and petiole, shape of ovipositor sheaths and their heavy pubescence. Davidian considered it as separate genus (Davidian 2007). However, since this species is a parasitoid of waxy root aphids, all those differences could represent adaptations to the specific underground ecological niche (Gärdenfors 1986). It is probable that *E. validus* and *E. helleni* separated from other species early in the evolution of this group, driven by a specialisation for a certain aphid host. The results of molecular analysis of nuclear EF1α grouped *E. lacertosus*, *E. helleni*, and *E. validus* into one clade, separating them from the rest of *plagiator* species.

The fact that revealing new cryptic species within large generalist groups is an ongoing process (Derocles 2016, Petrović et al. 2016) is once again proven by the description of the additional member of *plagiator* group, *E. hyadaphis*. The specimens reared from *Hyadaphis foeniculi* were collected across the Balkans during the period of fifteen years, from different plant hosts (*Lonicera xylosteum*, *Sanicula europaea*, and *Anthriscus sylvestris*), all common for this aphid species. *Ephedrus hyadaphidis* parasitoids were found in the woodland area on higher altitudes (524–1140 meters above sea level). This species might have been noticed earlier: in his revision Gärdenfors (1986) discusses an *Ephedrus* specimen collected in Italy, reared from *Hyadaphis foeniculi* on *Lonicera etrusca*, similar to *E. nacheri*, but with several morphological differences. Along with *E. lonicerae* and *E. tamaricis*, *E. hyadaphis* represents the third recently described species of *Ephedrus* distributed in the Balkan region. Furthermore, *E. persicae* is separated into two clades, one containing Mediterranean taxa, and the other taxa from the rest of Europe. The average genetic distance between these two clades is 2.5 %, suggesting that they have been evolving separately for some time. Thus, both clades require further taxonomic investigation. The richness of species that are distributed only in the Balkan region, as well as richness of haplotype numbers might imply that this area played a significant role for *Ephedrus* species as a refuge during the glacial periods.

*Ephedrus koponeni* is reported outside its known distribution, northern Europe (Finland and European part of Russia) (Halme 1992, Davidian 2018). One male and one female were reared from *Cinara* sp. on the Balkan pine, *Pinus peuce*, a relict and endemic species with a fragmented distribution in the Balkan Peninsula (in Serbia inhabiting only very southern part). It is possible that the specimens of this species across Europe are, when seldom collected, misidentified as *E. plagiator*. The other probability is that *E. koponeni* possesses an extremely fragmented distribution, restricted only to the secluded areas of its former species range.

The results of this study show close phylogenetic relationships among *E. koponeni*, *E. prociphili*, and *E. nacheri*. All three species are morphologically very similar to *E. plagiator*, distinguished from each other by subtle morphological differences and aphid host range (Gärdenfors 1986). While *E. koponeni* and *E. prociphili* are specialised to coniferous aphid species and *Prociphilus* sp., respectively, *E. nacheri* has a somewhat broader aphid host range, parasitising several species from Aphidini and Macrosiphini tribes. In Europe this species is most frequently reared from the genus *Hayhurstia* Del Guercio, 1917. These species might be quite young in the evolution line of the genus *Ephedrus*, just recently separated from the others in *plagiator* species group, which would explain lower genetic distances and great morphological similarity with *E. plagiator*. Furthermore, our study suggests that *E. cerasicola* (parasitoid of *Myzus* and *Dysaphis*) and *E. laevicollis* (parasitoid of *Chaetosiphon* Mordvilko, 1914), are closely related species.

The results of molecular analyses show that *Ephedrus* species, when multiple sequences for analysis are available, are rich in haplotype variety. *Ephedrus plagiator* is known as a broadly polyphagous species, distributed throughout Europe. Specimens analysed here were collected from various aphid hosts (*Aphis* Linnaeus, 1758, *Brachyunguis* Das, 1918, *Sitobion* Mordvilko, 1914, *Rhopalosiphum* Koch, 1854, *Macrosiphum* Oestlund, 1886, *Anoecia* Koch, 1857, *Linosiphon* Börner, 1944 and *Pterocomma* Buckton, 1879) and plants (*Poa*, *Dactylis*, *Malus*, *Tamarix*, *Triticum*, *Sitobion*, *Galium*, *Salix*, *Prenanthes*, *Abies*, *Vicia*, *Ranunculus*, *Prunus*, *Heracleum*); in 15 sequences eight haplotypes were identified, which all grouped together into one phylogenetic clade. Compared to *E. persicae*, where in 15 specimens 12 haplotypes (forming two clusters) were discovered (Petrović et al. 2016), the genetic variability within *E. plagiator* seems to be somewhat lower. *Ephedrus niger*, an Euroasian species mainly reared from *Uroleucon* Mordvilko, 1914 and *Macrosiphoniella* Del Guercio, 1911 aphid hosts (Gärdenfors 1986, Tomanović et al. 2009) is easily distinguished from the other *Ephedrus* species by dark to black body and longer F1 with a constriction in the basal third. Out of seven analysed specimens we identified seven different haplotypes which all grouped into one separate clade.

In summary, our results show that the phylogeny of *Ephedrus* is more complex than previously thought. It is important to note that European species of *Ephedrus* comprise less than a half of currently described species, so in order to get complete insight into the phylogenetic relationships among species and their evolution, further studies are needed.

## Supplementary Material

XML Treatment for Ephedrus hyadaphidis

XML Treatment for Fovephedrus

XML Treatment for Breviephedrus

XML Treatment for Ephedrus

## Supplementary materials

Supplementary material 1 *Ephedrus* specimens analysed in this study Data type: specimens data https://binary.pensoft.net/file/344722This dataset is made available under the Open Database License (http://opendatacommons.org/licenses/odbl/1.0/). The Open Database License (ODbL) is a license agreement intended to allow users to freely share, modify, and use this Dataset while maintaining this same freedom for others, provided that the original source and author(s) are credited.Korana Kocić, Andjeljko Petrović, Jelisaveta Čkrkić, Milana Mitrović, Željko Tomanović

Supplementary material 2 Estimates of evolutionary divergence between sequences Data type: phylogenetic data https://binary.pensoft.net/file/344723This dataset is made available under the Open Database License (http://opendatacommons.org/licenses/odbl/1.0/). The Open Database License (ODbL) is a license agreement intended to allow users to freely share, modify, and use this Dataset while maintaining this same freedom for others, provided that the original source and author(s) are credited.Korana Kocić, Andjeljko Petrović, Jelisaveta Čkrkić, Milana Mitrović, Željko Tomanović
